# A novel and two recurrent UMOD mutations in autosomal dominant tubulointerstitial kidney disease (ADTKD): a case series and literature review

**DOI:** 10.1080/0886022X.2026.2653343

**Published:** 2026-04-09

**Authors:** Yedong Yu, Jianguang Gong, Lin Liu, Lina Shao, Xiaogang Shen, Bin Zhu

**Affiliations:** Urology and Nephrology Center, Department of Nephrology, Zhejiang Provincial People’s Hospital, Affiliated People’s Hospital, Hangzhou Medical College, Hangzhou, Zhejiang, China

**Keywords:** UMOD, autosomal dominant tubulointerstitial kidney disease, ADTKD-UMOD, next-generation sequencing, mutation spectrum, genetic diagnosis

## Abstract

Autosomal dominant tubulointerstitial kidney disease (ADTKD) is a rare hereditary disorder characterized by slowly progressive loss of kidney function with absent or mild proteinuria and tubulointerstitial fibrosis. Mutations in the *UMOD* gene represent one of the primary genetic etiologies of ADTKD. This study presented the clinical data and genetic variant interpretation of three ADTKD patients diagnosed *via* a next-generation sequencing (NGS)-based diagnostic workflow at the Department of Nephrology, Zhejiang Provincial People’s Hospital, along with a review of relevant literature. Genetic analysis revealed three heterozygous UMOD variants: a novel p. Tyr559Cys, a variant previously recorded in databases (p. Ala500Val) but with limited clinical details, and a previously published variant (p. Leu66Pro) in a new family. This report contributes to expanding the mutation spectrum of *UMOD* and highlights its phenotypic variability, thereby enhancing clinical recognition and promoting timely diagnosis.

## GRAPHICAL ABSTRACT

**Figure:**
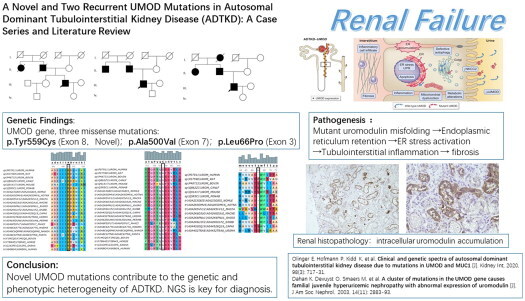

## Introduction

ADTKD is a rare, genetically heterogeneous disorder characterized by progressive renal dysfunction with absent or mild proteinuria and hematuria. Since the Kidney Disease: Improving Global Outcomes (KDIGO) initiative formalized its diagnostic framework, ADTKD has been clearly defined, genotyped, and recognized as a clinically significant entity within the nephrology community. To date, six pathogenic genes—*UMOD*, *MUC1*, *REN*, *HNF1B*, *SEC61A1*, and *DNAJB11*—have been implicated in ADTKD pathogenesis. Among international cohorts, ADTKD-*UMOD* represents the most prevalent subtype, accounting for approximately 37.1% of cases [1]. The hallmark features of ADTKD-*UMOD* include early-onset hyperuricemia or gout. Compared to ADTKD-*MUC1*, ADTKD-*UMOD* demonstrates an earlier clinical onset but a slower progression to end-stage kidney disease (ESKD) [1]. The age of ESKD onset in ADTKD-*UMOD* exhibits considerable heterogeneity, ranging from 18 to 87 years, with male patients showing a significantly higher risk of disease progression. Histologically, tubulointerstitial fibrosis is a universal feature of ADTKD. Given the nonspecific clinical presentation, diagnosing ADTKD remains challenging in clinical practice, necessitating reliance on family history and confirmatory genetic testing. In this study, we report three genetically confirmed ADTKD-*UMOD* cases identified *via* NGS at our department, supplemented by a comprehensive review of recent advances in the field. Our findings aim to enhance clinical awareness and inform optimal management strategies for this underrecognized disorder.

## Case report

### Patient 1

#### General information

**Figure 1.**
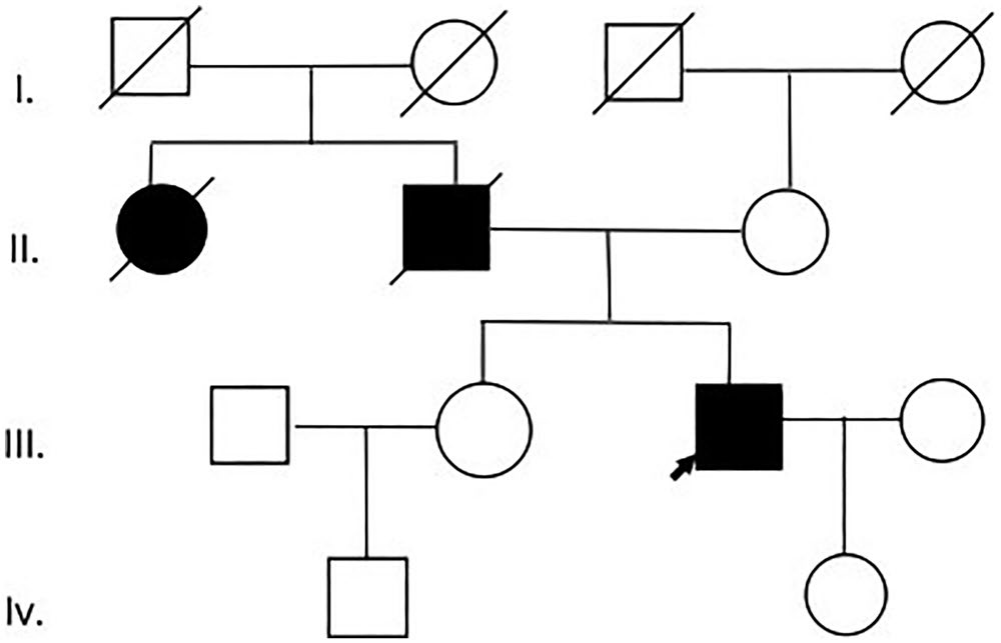
Patient 1: Family pedigree.

The proband, a 60-year-old male, presented to our department on January 17, 2024, with a 5-year history of elevated serum creatinine (110–160 μmol/L) and persistent proteinuria (1–2+). His medical history included long-standing hypertension and gout (duration >10 years). Notably, his family history was significant, with both his father and maternal aunt succumbing to uremia (Figure 1). Physical examination was unremarkable.

#### Laboratory examinations (Table 1)

The laboratory workup revealed 2+ proteinuria without hematuria, with elevated 24-h urine protein excretion of 1.1 g/24h (normal range: 0–0.15 g/24h). Serum biochemistry demonstrated normal albumin (40.5 g/L; reference 40–55 g/L), elevated creatinine (153.4 μmol/L; reference 57–111 μmol/L), and normal uric acid levels (221 μmol/L; reference 208–428 μmol/L^1^). Immunological profiles showed normal IgG/IgA/IgM, C3/C4 complement levels, and negative autoantibodies (ANA, dsDNA, anti-PLA2R, ANCA). Infectious disease screening was negative for HBV, HCV, HIV, syphilis, HSV, and CMV, with normal hematologic, coagulation, and thyroid function parameters. Ophthalmologic examination demonstrated no anterior lenticonus or perimacular dot/fleck retinopathy, while audiometry showed no bilateral high-frequency sensorineural hearing loss. Renal ultrasound revealed normal-sized kidneys with altered parenchymal echogenicity and a 2.0 × 1.6 cm right renal cyst.

#### Pathologic examination

**Figure 2.**
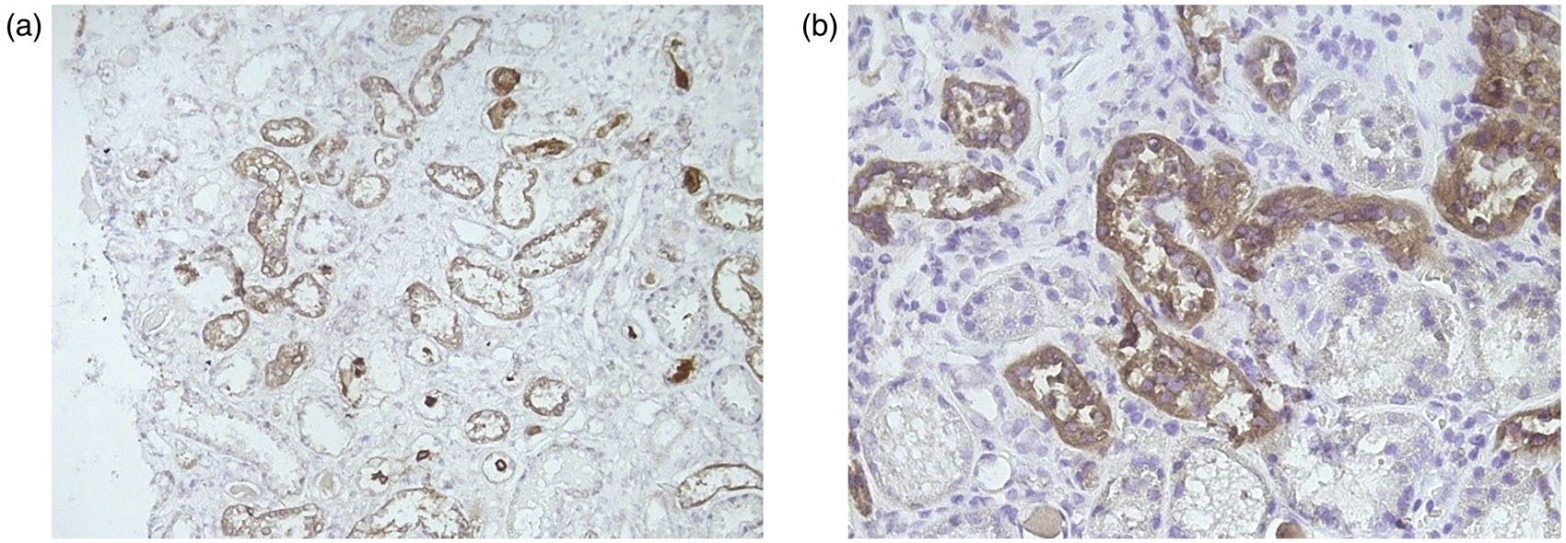
Renal histopathology of Patient 1 showing intracellular uromodulin accumulation.

On light microscopy, histopathological examination showed tubulointerstitial fibrosis, tubular atrophy with cystic dilatation, and global glomerulosclerosis (53%). Immunofluorescence was negative for immunoglobulins and complement. Immunohistochemistry using anti-*UMOD* antibody (EPR20071; Abcam) revealed abnormal intracellular accumulation of mutant uromodulin in distal tubule epithelial cells (Figure 2a and b). Electron microscopy revealed one obsolescent glomerulus with characteristic alterations: capillary endothelial vacuolization, segmental GBM thickening (250–750 nm), and diffuse podocyte foot process effacement (>80%). The mesangium showed mild hypercellularity with rare electron-dense deposits. Tubulointerstitial findings included tubular epithelial vacuolization, focal basement membrane thickening, and mild inflammatory infiltrates. Vascular changes were limited to occasional capillary erythrocyte aggregates.

#### Gene mutation examination

**Figure 3.**
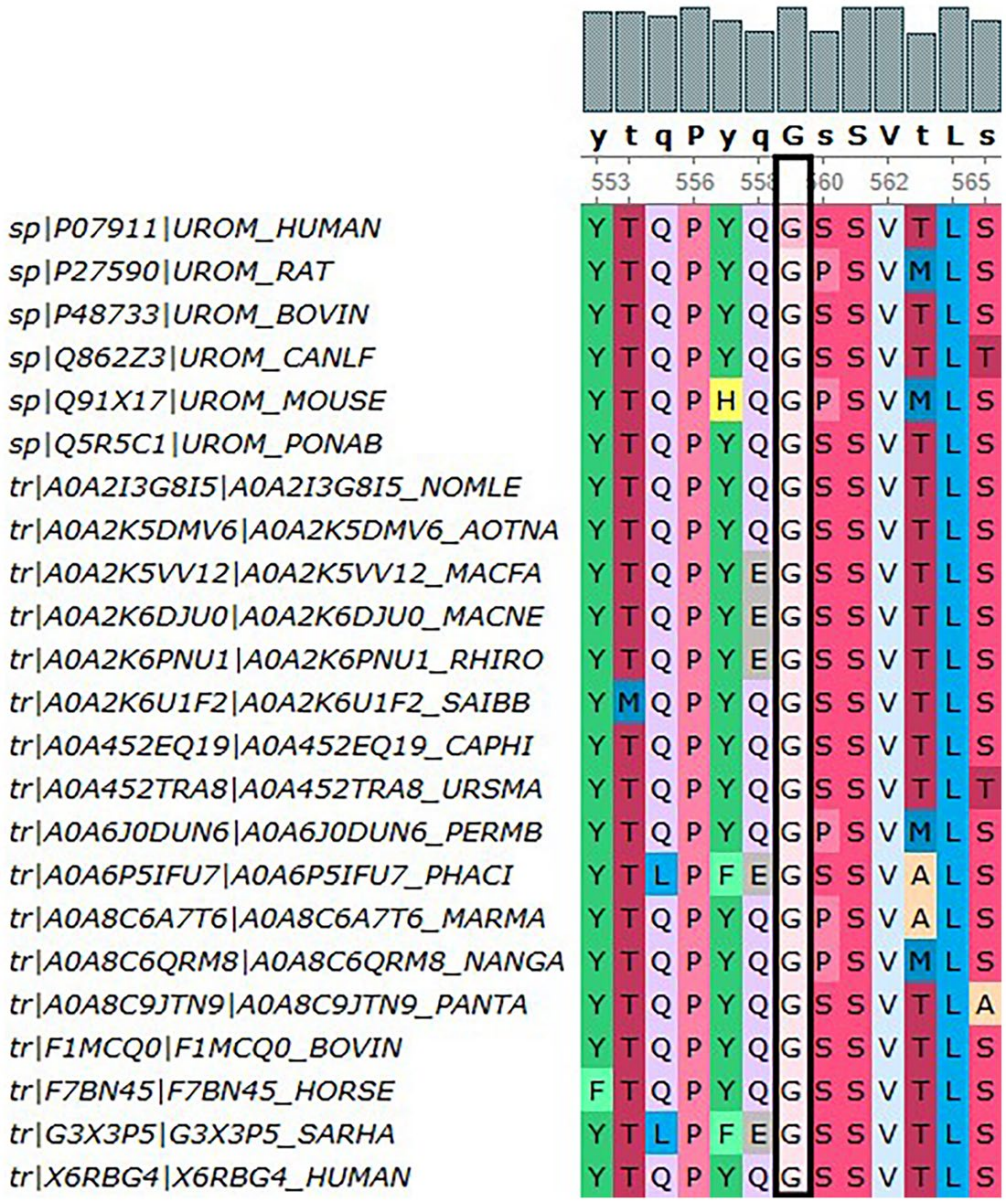
Patient 1: The tyrosine residue at position 559 in UMOD shows high cross-species conservation (p. Tyr559Cys).

NGS revealed a novel heterozygous missense mutation in *UMOD* (NM_003361.4: c.1676A > G, p. Tyr559Cys), located within exon 8. This variant was absent from all major genomic databases (e.g. gnomAD, NCBI、OMIM, and others) and had been submitted to ClinVar with accession number RCV006253441.1. Bioinformatics analysis demonstrated that the affected tyrosine residue at position 559 was evolutionarily conserved across species (Figure 3, Ugene analysis).

#### Treatment and follow-up

The patient was diagnosed with chronic kidney disease (CKD) stage 3. The patient was managed with a comprehensive therapeutic regimen including valsartan (for proteinuria control), empagliflozin (for kidney protection), febuxostat (for hyperuricemia management), and sodium bicarbonate (for urine alkalization) (Table 1). At the 1-year follow-up (January 22, 2025), laboratory evaluation demonstrated stable renal function (serum creatinine 155.8 μmol/L) with significantly improved proteinuria (trace [1+] (near-normal) on urinalysis; urine protein-to-creatinine ratio 0.29).

**Table 1.** Clinical information and treatment of the patients.

Item	Patient 1	Patient 2	Patient 3	Normal range
Hemoglobin (g/L)	129	132	124	130–175
Albumin (g/L)	40.5	46.1	44.9	40–55
White blood cells (/L)	8.21 × 10^9^	7.92 × 10^9^	9.18 × 10^9^	3.5–9.5 × 10^9^
Serum creatinine (μmol/L)	153.4	120	142.5	57–111
Urea (mmol/L)	6.71	7.77	6.5	3.6-9.5
eGFR (mL/min/1.73 m²)	42.11	63.28	52.6	G1(≥90) G2 (60–89) G3a (45–59) G3b (30–44) G4 (15–29) G5 (<15)
Uric acid (μmol/L)	221	458	531	Male 208–428; Female 155–357
Na+ (mmol/L)	138.5	139.5	139.8	137–147
K+(mmol/L)	4.54	4.35	3.97	3.5–5.5
Urine red blood cell (/uL)	0.6	36.2	2.8	0–18
24 h urine protein (g)	1.1	0.085	0.163	0–0.15
Treatment	BP control: Valsartan 80 mg once daily	Hyperuricemia control: Febuxostat 40 mg once daily	Hyperuricemia control: Febuxostat 40 mg once daily	
	Antiproteinuric effect: empagliflozin 10 mg once daily			
	Hyperuricemia control: Febuxostat 20 mg once daily; Sodium bicarbonate: 500 mg three times a day			

### Patient 2

#### General information

**Figure 4.**
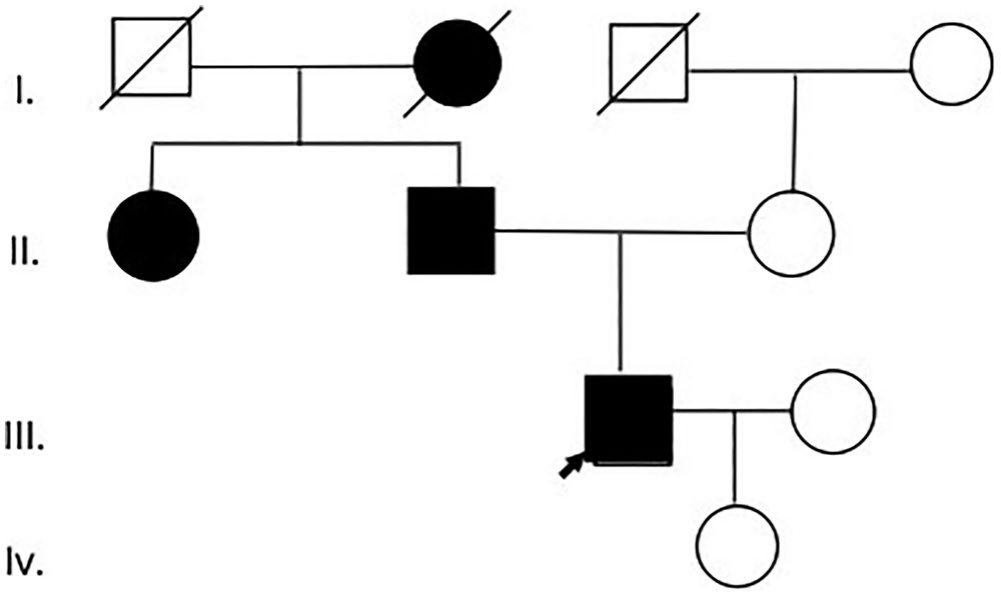
Patient 2: Family pedigree.

A 36-year-old male proband presented to our department on April 7, 2024, with a 3-year history of persistently elevated serum creatinine and abnormal urinalysis. Serum creatinine fluctuated between 119 and 129 μmol/L over the past 3 years. Urinalysis demonstrated microscopic hematuria (occult blood 2+) without proteinuria. The patient had no personal history of hypertension or gout. Family history was significant for chronic kidney disease, including: (1) father with end-stage renal disease (status post renal transplantation), (2) maternal aunt with stage 4 chronic kidney disease (5-year duration), and (3) paternal grandmother who succumbed to uremia (Figure 4). Physical examination was unremarkable.

#### Laboratory examinations (Table 1)

Laboratory evaluation revealed microscopic hematuria (36.2 RBCs/μL) without proteinuria, with normal 24-h urinary protein excretion (0.085 g). Serum biochemistry showed normal albumin (40.8 g/L), mildly elevated creatinine (120 μmol/L), and hyperuricemia (458 μmol/L). Comprehensive workup excluded secondary causes of nephropathy. Renal ultrasound revealed normal-sized kidneys with altered parenchymal echogenicity and a 2.0 × 0.5 cm right renal cyst.

#### Gene mutation examination

**Figure 5.**
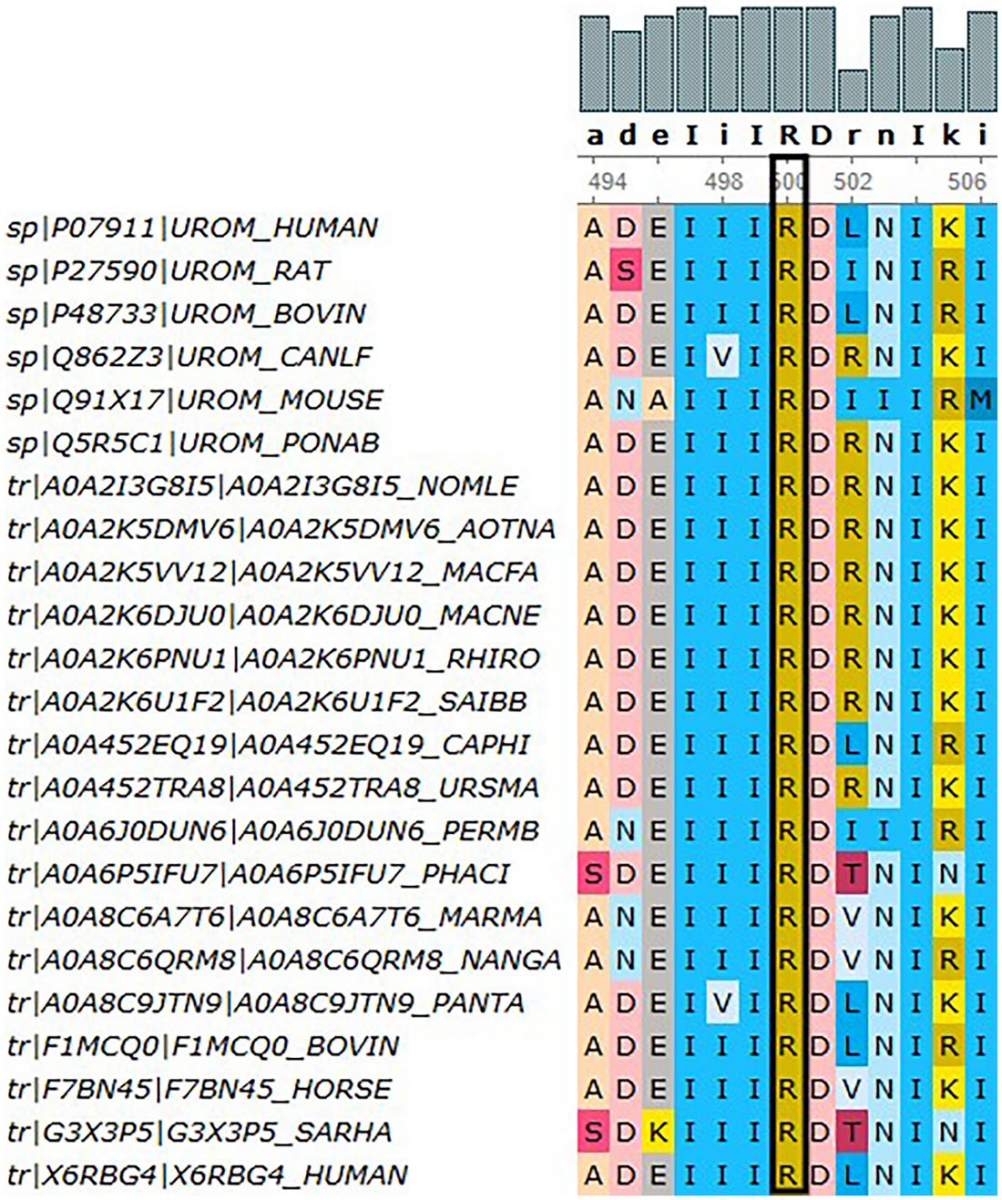
Patient 2: The alanine residue at position 500 in UMOD shows high cross-species conservation (p. Ala500Val).

NGS analysis identified a heterozygous *UMOD* mutation (NM_003361.4: c.1499C > T: p. Ala500Val), located within exon 7. The affected alanine residue at position 500 demonstrated remarkable evolutionary conservation across species (Figure 5). This variant was recorded in ClinVar under accession number RCV005019788.2. While this variant had been submitted to the ClinVar database, it had not been comprehensively described in the peer-reviewed literature. Our case provided detailed clinical and phenotypic information for this variant.

#### Treatment and follow-up

The patient was diagnosed with CKD stage 2. The patient was managed with febuxostat for hyperuricemia control (Table 1). At the 6-month follow-up (November 12, 2024), laboratory assessment demonstrated stable renal function (serum creatinine 120 μmol/L) with resolution of proteinuria (urine dipstick negative) and minimal microscopic hematuria (20 RBCs/μL).

### Patient 3

#### General information

**Figure 6.**
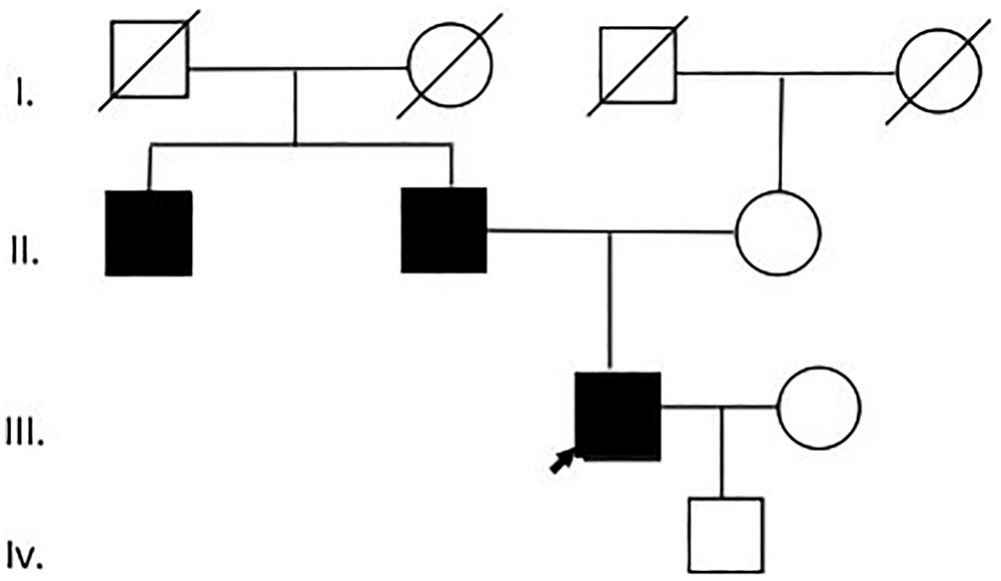
Patient 3: Family pedigree.

A 41-year-old male proband presented to our department on June 23, 2023, with a 5-year history of persistently elevated serum creatinine and abnormal urinalysis findings. His serum creatinine fluctuated between 110 and 142 μmol/L over the past 5 years. Laboratory evaluation revealed trace proteinuria without hematuria. The patient had no personal history of hypertension or gout. His father and uncle both developed ESRD (see pedigree, Figure 6). Physical examination was unremarkable.

#### Laboratory examinations (Table 1)

Laboratory evaluation demonstrated trace proteinuria (24-h urinary protein excretion: 0.163 g) without hematuria. Serum biochemistry revealed normal albumin levels (40.1 g/L) with elevated serum creatinine (142.5 μmol/L) and marked hyperuricemia (531 μmol/L). Comprehensive workup excluded secondary causes of nephropathy. Renal ultrasound revealed normal-sized kidneys with altered parenchymal echogenicity and a 1.4 × 1.4 cm right renal cyst.

#### Gene mutation examination

**Figure 7.**
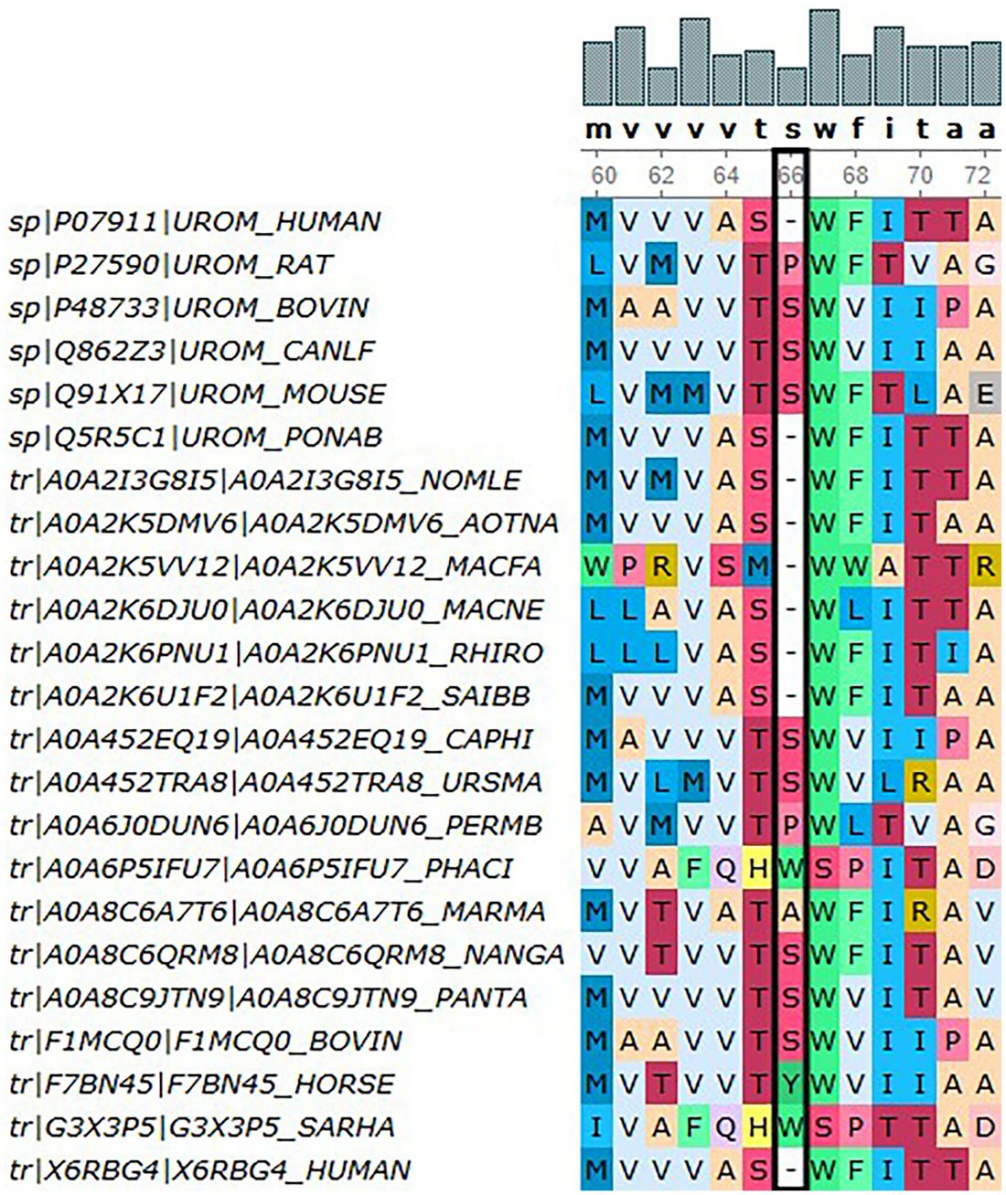
Patient 3: The leucine residue at position 66 in UMOD shows high cross-species conservation (p. Leu66Pro).

NGS analysis identified a heterozygous *UMOD* mutation (NM_003361.4: c.197T > C: p. Leu66Pro), located within exon 3. The affected leucine residue at position 66 demonstrated exceptional evolutionary conservation across vertebrate species (Figure 7). This variant had been submitted to ClinVar with accession number RCV002251377.14. Although this variant had been previously reported in a Chinese family with ADTKD [2], Our independent identification of this variant in a new family confirmed its pathogenicity and allowed for further observation of its phenotypic expression.

#### Treatment and follow-up

The patient was diagnosed with CKD stage 3. The patient was managed with febuxostat, combined with nephroprotective agents (Table 1). At the 6-month follow-up (September 12, 2024), laboratory assessment demonstrated stable renal function (serum creatinine: 99 μmol/L) without proteinuria and hematuria.

## Discussion and conclusions

We presented three male cases of ADTKD diagnosed at our department, showing varying degrees of renal dysfunction with predominant tubulointerstitial injury. Based on clinical and histopathological findings combined with positive family history, hereditary kidney disease was strongly suspected. Definitive diagnosis of ADTKD-*UMOD* was established through a comprehensive NGS-based diagnostic workflow. Among the three variants we report, only p. Tyr559Cys (exon 8) represented a novel mutation, absent from public databases including gnomAD, ClinVar, and other literature, but now submitted to ClinVar (accession: RCV006253441.1). The p. Ala500Val (exon 7) variant was recorded in ClinVar (accession: RCV005019788.2) but lacks a detailed clinical description in the literature. Our report provided valuable phenotypic data for this variant, noting the presence of hematuria in our patient. Finally, the p. Leu66Pro (exon 3) variant was previously documented in a 44-year-old male Chinese patient who presented with renal insufficiency and hyperuricemia (Xia et al. 2015) [2]. Our independent identification of this variant in a younger proband (41 years old) with similarly marked hyperuricemia provides robust confirmation of its pathogenicity. Furthermore, characterizing this recurrent mutation in a new pedigree strengthens the evidence for its consistent association with early-onset hyperuricemia and contributes to a more precise genotype-phenotype correlation.

Following the ACMG guidelines, we classified all three variants as Likely Pathogenic (Table 2). Key evidence included the absence of these variants in the gnomAD population database (PM2), their co-segregation with disease in families (PP1), deleterious predictions from evolutionary conservation and bioinformatic tools (PP3), and their occurrence in patients with a highly specific ADTKD phenotype (PP4). These affected amino acid residues all demonstrate remarkable evolutionary conservation across species. Notably, our three cases exhibited considerable phenotypic variability despite all carrying pathogenic *UMOD* mutations. Patient 3 (p. Leu66Pro) presented with marked hyperuricemia at a young age, while Patient 1 (p. Tyr559Cys) had more pronounced proteinuria, and Patient 2 (p. Ala500Val) presented with hematuria. This observed heterogeneity underscores the challenges in predicting disease course based on genotype alone and highlights the complex relationship between *UMOD* mutations and their clinical manifestations. Consequently, our findings reinforce the importance of genetic counseling for affected families to communicate the variable expressivity, inheritance risk, and available management options (Table 3).

**Table 2.** ACMG classification and supporting evidence for the identified UMOD variants.

Variant (Nucleotide)	Variant (Protein)	ACMG Classification	Supporting Evidence Codes
c.1676A > G	p. Tyr559Cys	Likely Pathogenic	PM2, PP1, PP3, PP4
c.1499C > T	p. Ala500Val	Likely Pathogenic	PM2, PP1, PP3, PP4
c.197T > C	p. Leu66Pro	Likely Pathogenic	PM1, PM2, PP1, PP3, PP4

abbreviation: PM1: Located in a mutational hot spot and/or critical and well-established functional domain; PM2: Absent from controls in population databases; PP1: Co-segregation with disease in multiple affected family members; PP3: Multiple lines of computational evidence support a deleterious effect; PP4: Patient’s phenotype is highly specific for the disease.

**Table 3.** Cross-sectional comparison of clinical severity by UMOD mutation location.

Parameter	Exon3 (p. Leu66Pro)	Exon7 (p. Ala500Val)	Exon8 (p. Tyr559Cys)	Comment/Trend
Age (years)	41	36	60	Exon 8 case was older at diagnosis.
Serum Creatinine (μmol/L)	142.5	120	153.4	Exon 8 > Exon 3 > Exon 7
eGFR (mL/min/1.73 m²)	52.6	63.3	42.1	Exon 8 < Exon 3 < Exon 7
Uric Acid (μmol/L)	531	458	221	Markedly elevated in Exon 3 case
24h Proteinuria (g)	0.163	0.085	1.1	Pronounced in Exon 8 case
Hematuria	Absent	Present	Absent	Unique to Exon 7 case
CKD Stage	Stage 3	Stage 2	Stage 3	Exon 7 mildest stage

eGFR: estimated glomerular filtration rate; CKD: chronic kidney disease.

ADTKD is now recognized as the third most common inherited cause of kidney disease worldwide, following autosomal dominant polycystic kidney disease (ADPKD) and Alport syndrome. ADTKD-*UMOD* represents the most common ADTKD subtype [1]. The *UMOD* gene, located at chromosome 16p12.3, encodes uromodulin (Tamm-Horsfall protein), the most abundant urinary protein under physiological conditions. Predominantly expressed in the thick ascending limb of Henle, uromodulin is synthesized by thick ascending limb epithelial cells and released into tubular lumen following serine protease processing [3]. Its multifunctional roles include salt handling, infection/stone prevention, and immunomodulation [4–6].

The pathogenesis of renal injury in ADTKD-*UMOD* is primarily mediated through the following mechanisms: mutant UMOD proteins encoded by pathogenic *UMOD* variants undergo misfolding and subsequent retention within the endoplasmic reticulum (ER) of thick ascending limb of Henle (TALH) cells. This aberrant intracellular accumulation triggers ER stress responses, ultimately leading to tubulointerstitial inflammation and progressive fibrosis [7–9].

ADTKD-*UMOD* primarily affects tubulointerstitial compartments, featuring slowly progressive renal dysfunction with absent or minimal proteinuria/hematuria, often accompanied by hyperuricemia/gout. ADTKD typically presents with an insidious onset, and most of its clinical manifestations—such as decreased glomerular filtration rate (GFR) or hyperuricemia - are nonspecific and commonly observed in CKD of other etiologies. This lack of disease-specific clinical features makes ADTKD particularly challenging to diagnose.

The renal pathology in ADTKD patients typically demonstrates nonspecific findings, including tubulointerstitial fibrosis, tubular atrophy, and thickening of tubular basement membranes, with glomeruli appearing either normal or showing sclerosis. Immunohistochemistry with anti-*UMOD* antibodies demonstrates substantial accumulation of *UMOD* protein, which is visualized more distinctly by immunofluorescence staining [10]. Light microscopic identification of abnormal uromodulin accumulation within tubular epithelial cells remains technically challenging in renal biopsy specimens. However, current literature identifies periodic acid-Schiff (PAS) staining as the most sensitive histological method for *UMOD* detection, with studies reporting identifiable pathological accumulation in 85% of genetically confirmed ADTKD-*UMOD* cases [10,11]. The detection sensitivity of aggregated *UMOD* deposits appears to correlate positively with glomerular filtration rate (GFR), suggesting better diagnostic yield of renal biopsy in ADTKD-*UMOD* patients with preserved renal function [12].

Genetic testing now serves as the diagnostic gold standard for ADTKD, as established by the 2015 KDIGO consensus guidelines (Eckardt et al. [13]). A definitive ADTKD diagnosis requires: (1) identification of a pathogenic variant in known ADTKD-associated genes (*UMOD, MUC1, REN*, etc.) in the proband or affected relative, or (2) clinical and histopathological evidence of autosomal dominant CKD in ≥1 family member. Notably, approximately 15-20% of ADTKD-*UMOD* cases occur sporadically due to novel mutations, meaning the absence of family history cannot exclude this diagnosis. Bolle et al. [14] reported that approximately 10% of ADTKD-*UMOD* cases result from novel mutations. In patients with variants of uncertain significance (VUS) in *UMOD*, the observation of abnormal uromodulin accumulation in renal tissue may serve as supporting evidence for pathogenicity, thereby strengthening the genetic diagnosis of ADTKD-*UMOD*.

The *UMOD* gene comprises 11 exons, with exons 2–11 encoding uromodulin. Initial work by Williams et al. [15] identified mutations in exons 3–5 and 7. Subsequent research had revealed that >95% of *UMOD* mutations cluster in exons 3 and 4, where missense mutations disrupt disulfide bond formation between cysteine residues. A comprehensive overview of the mutational landscape was provided in Supplementary Table S1. Notably, our patient 1 carried a rare exon 8 mutation, echoing the first reported Chinese case of an exon 8 variant (c.1648G > A, p. V550I) by Jing Yang et al. [16]. That pediatric case presented with hematuria and had a family history of ESRD at age 29 in the father, with diagnosis ultimately confirmed by whole-exome sequencing as ADTKD-*UMOD*. The inclusion of our p. Tyr559Cys variant in Supplementary Table S1 further enriches the spectrum of rare mutations documented in exon 8.

ADTKD-*UMOD* remains a rare disorder in clinical practice, with fewer than 2,000 reported cases worldwide to date. Its nonspecific clinical and histopathological features underscore the necessity of genetic testing as the diagnostic gold standard. Given the autosomal dominant inheritance pattern, affected parents have a 50% probability of transmitting the disease to their offspring. Prenatal genetic testing enables early diagnosis, while comprehensive screening in suspected cases facilitates informed reproductive counseling [17].

Currently, there exists no specific therapy for ADTKD, with management primarily focusing on supportive care and treatment of CKD-related complications. ADTKD-*UMOD* patients may progress to ESKD between 20 and 70 years of age. Despite advances in genetic diagnosis, ADTKD management remains limited to renal replacement therapy (dialysis or transplantation); no targeted treatment options with demonstrated prognostic benefits are currently available.

With the growing complexity of genetic interpretation in ADTKD, particularly for novel or ultra-rare *UMOD* variants, AI-based pathogenicity prediction models offer a transformative approach to variant classification [18,19]. Tools such as Alpha Missense [20], EVE (Evolutionary model of Variant Effect) [21], and similar deep learning frameworks integrate evolutionary conservation, protein structure, and functional annotations to infer pathogenic potential with high precision. These AI tools could be instrumental in prioritizing VUS in *UMOD* for subsequent functional validation and in delineating the underlying mechanisms of genotype–phenotype correlations in ADTKD.

This study has several limitations. A major limitation is the absence of functional assays to validate the identified *UMOD* mutations. While evidence from clinical segregation, conservation data, and histopathological findings strongly suggests pathogenicity, functional studies will be essential to definitively confirm their deleterious impact on uromodulin biology.

Future directions of the study should prioritize mechanistic and translational advances to bridge the current gap between genetic diagnosis and targeted therapy in ADTKD-UMOD. First, functional validation of newly identified and ultra-rare UMOD variants, particularly those outside classical mutational hotspots such as exon 8, using *in vitro* cellular models, patient-derived tubular organoids, and *in vivo* knock-in systems is essential to clarify their effects on uromodulin folding, endoplasmic reticulum stress, and downstream inflammatory and fibrotic pathways. Second, integrative genotype–phenotype studies in larger, multicenter cohorts are needed to refine prognostic stratification, identify modifiers of disease onset and progression, and explain the marked phenotypic heterogeneity observed among patients with different UMOD mutations. Third, the incorporation of advanced AI-based variant interpretation tools combined with structural modeling and longitudinal clinical data may improve pathogenicity classification of variants of uncertain significance and support precise genetic counseling. Finally, future research should explore targeted therapeutic strategies aimed at reducing endoplasmic reticulum stress, enhancing mutant protein clearance, or modulating tubular injury pathways, thereby moving ADTKD-UMOD management beyond supportive care toward mechanism-based interventions.

In summary, ADTKD-*UMOD* presents with insidious onset and exhibits highly variable clinical and pathological manifestations that lack specificity, often leading to missed or incorrect diagnoses. In clinical practice, obtaining a detailed family history is paramount, while use of next-generation sequencing (NGS) as a diagnostic gold standard is for both diagnosis and subtyping. Consequently, for patients presenting with clinical features suggestive of ADTKD, early genetic screening for causative mutations is crucial—not only for accurate diagnosis and timely treatment but also for facilitating informed reproductive decisions and genetic counseling.

## Supplementary Material

Supplemental Material

## Data Availability

The authors confirm that the data supporting the findings of this study are available within the article and its supplementary materials.

## Note

1 Institutional reference range for uric acid: male 208–428 μmol/L, female 155–357 μmol/L.
