# Supplementary material for: A novel and two recurrent UMOD mutations in autosomal dominant tubulointerstitial kidney disease (ADTKD): a case series and literature review
Source: Ren Fail. 2026 Apr 9;48(1):2653343. doi: 10.1080/0886022X.2026.2653343 (PMC13072683; doi:10.1080/0886022X.2026.2653343)
Supplement: Supplemental Material [file IRNF_A_2653343_SM5051.docx]

**Supplementary Table S1.** Summary of Representative UMOD Mutations Grouped by Exon

| Exon | DNA change | **Protein Change** | Manifestation | PMID |
| --- | --- | --- | --- | --- |
| Exon 3 | c.95G>A | p. Cys32Tyr | NA | 32305225 |
| Exon 3 | c.95G>C | p. Cys32Ser | NA | NA |
| Exon 3 | c.96C>G | p. Cys32Trp | NA | 32305225 |
| Exon 3 | c.100G>A | p. Glu34Lys | NA | 32305225 |
| Exon 3 | c.104G>A | p. Cys35Tyr | HUA, CKD | 33384418 |
| Exon 3 | c.113A>T | p. Asn38Ile | HUA, CKD | 33384418 |
| Exon 3 | c.115G>A | p. Ala39Thr | NA | 32305225 |
| Exon 3 | c.121T>C | p. Cys41Arg | HUA, CKD | 36290580 |
| Exon 3 | c.155G>C | p. Cys52Ser | NA | 32305225 |
| Exon 3 | c.155G>A | p. Cys52Tyr | NA | 32305225 |
| Exon 3 | c.163G>A | p. Gly55Ser | NA | 32305225 |
| Exon 3 | c.172G>T | p. Gly58Cys | NA | NA |
| Exon 3 | c.175G>T | p. Asp59Tyr | NA | NA |
| Exon 3 | c.176A>C | p. Asp59Ala | NA | 32305225 |
| Exon 3 | c.179G>A | p. Gly60Asp | HUA, CKD | 36290580 |
| Exon 3 | c.188G>C | p. Cys63Ser | NA | 32305225 |
| Exon 3 | c.193G>A | p. Asp65Asn | NA | NA |
| Exon 3 | c.197T>C | p. Leu66Pro | HUA, CKD | This study |
| Exon 3 | c.202G>A | p. Glu68Lys | NA | 32305225 |
| Exon 3 | c.205T>C | p. Cys69Arg | NA | 32305225 |
| Exon 3 | c.228C>G | p. Asn76Lys | NA | 32305225 |
| Exon 3 | c.229T>G | p. Cys77Gly | NA | 32305225 |
| Exon 3 | c.229T>C | p. Cys77Arg | NA | 32305225 |
| Exon 3 | c.230G>A | p. Cys77Tyr | NA | 32305225 |
| Exon 3 | c.247T>C | p. Cys83Arg | NA | 32305225 |
| Exon 3 | c.247T>G | p. Cys83Gly | NA | NA |
| Exon 3 | c.255C>G | p. Asn85Lys | NA | 32305225 |
| Exon 3 | c.254A>G | p. Asn85Ser | NA | 32305225 |
| Exon 3 | c.263G>T | p. Gly88Val | HUA, CKD | 31412356 |
| Exon 3 | c.272delC | p. Ser91 | HUA, CKD | 25904457 |
| Exon 3 | c.274T>G | p. Cys92Gly | NA | 32305225 |
| Exon 3 | c.274T>C | p. Cys92Arg | NA | 32305225 |
| Exon 3 | c.275G>A | p. Cys92Tyr | NA | 32305225 |
| Exon 3 | c.278delT | p. Val93 | NA | 32305225 |
| Exon 3 | c.280T>C | p. Cys94Arg | HUA, CKD | 2022 |
| Exon 3 | c.278_289delTCTGCCCCG AAG insCCGGCTCCT | p. Val93_Gly97delinsAlaAlaSerCys | NA | 32305225 |
| Exon 3 | c.282C>G | p. Cys94Trp | NA | 32305225 |
| Exon 3 | c.287G>C | p. Cys96Ser | HUA, CKD | 37906055 |
| Exon 3 | c.307G>T | p. Gly103Cys | NA | 32305225 |
| Exon 3 | c.317G>T | p. Cys106Phe | NA | 32305225 |
| Exon 3 | c.316T>G | p. Cys106Gly | NA | 32305225 |
| Exon 3 | c.317G>A | p. Cys106Tyr | NA | 32305225 |
| Exon 3 | c.326T>A | p. Val109Glu | HUA, HTN, CKD | 24096149 |
| Exon 3 | c.334T>G | p. Cys112Gly | HUA, CKD | 33384418 |
| Exon 3 | c.334T>C | p. Cys112Arg | NA | 32305225 |
| Exon 3 | c.336C>G | p. Cys112Trp | NA | NA |
| Exon 3 | c.358T>C | p. Cys120Arg | NA | 32305225 |
| Exon 3 | c.359G>C | p. Cys120Ser | NA | NA |
| Exon 3 | c.376T>C | p. Cys126Arg | NA | 32305225 |
| Exon 3 | c.376T>A | p. Cys126Ser | NA | NA |
| Exon 3 | c.377G>A | p. Cys126Tyr | HUA, CKD | NA |
| Exon 3 | c.383A>G | p. Asn128Ser | NA | 32305225 |
| Exon 3 | c.403T>G | p. Cys135Gly | NA | NA |
| Exon 3 | c.404G>T | p. Cys135Phe | NA | 32305225 |
| Exon 3 | c.403T>A | p. Cys135Ser | NA | 32305225 |
| Exon 3 | c.405C>G | p. Cys135Trp* | NA | 32305225 |
| Exon 3 | c.404G>A | p. Cys135Tyr | NA | 32305225 |
| Exon 3 | c.428G>T | p. Cys143Phe | NA | NA |
| Exon 3 | c.442T>C | p. Cys148Arg | NA | 32305225 |
| Exon 3 | c.442T>A, c.443G>C | p. Cys148Ser | NA | 32305225 |
| Exon 3 | c.443G>A | p. Cys148Tyr | NA | 32305225 |
| Exon 3 | c.449G>C | p. Cys150Ser | NA | 32305225 |
| Exon 3 | c.459C>T | p. Gly153Gly | NA | 32305225 |
| Exon 3 | c.478G>C | p. Asp160His | NA | 32305225 |
| Exon 3 | c.483C>G | p. Cys161Trp | NA | NA |
| Exon 3 | c.509G>A | p. Cys170Tyr | NA | 32305225 |
| Exon 3 | c.514G>C | p. Asp172His | NA | 32305225 |
| Exon 3 | c.518C>T | p. Pro173Leu | NA | 32305225 |
| Exon 3 | c.520T>C | p. Cys174Arg | NA | 32305225 |
| Exon 3 | c.529_555del | p. His177_Arg185del | NA | 32305225 |
| Exon 3 | c.533G>C | p. Arg178Pro | NA | 32305225 |
| Exon 3 | c.539T>C | p. Leu180Pro | NA | 32305225 |
| Exon 3 | c.538C>G | p. Leu180Val | NA | 32305225 |
| Exon 3 | c.552G>C | p. Trp184Cys | NA | 32305225 |
| Exon 3 | c.553C>T | p. Arg185Cys | NA | 32305225 |
| Exon 3 | c.553C>A | p. Arg185Ser | NA | 32305225 |
| Exon 3 | c.553C>G | p. Arg185Gly | NA | 32305225 |
| Exon 3 | c.554G>A | p. Arg185His | HUA, CKD | 31412356 |
| Exon 3 | c.563_661del | p. Glu188_Leu221del | NA | 32305225 |
| Exon 3 | c.574G>C | p. Gly192Arg | NA | NA |
| Exon 3 | c.584G>A | p. Cys195Tyr | NA | 32305225 |
| Exon 3 | c.585_586CG>TA | p. Asp196Asn | NA | 32305225 |
| Exon 3 | c.586G>T | p. Asp196Tyr | NA | 32305225 |
| Exon 3 | c.586G>A | p. Asp196Asn | HUA, CKD | NA |
| Exon 3 | c.601G>C | p. Gly201Arg | NA | NA |
| Exon 3 | c.602G>A | p. Gly201Asp | NA | 32305225 |
| Exon 3 | c.606G>T | p. Trp202Cys | NA | 32305225 |
| Exon 3 | c.605G>C | p. Trp202Ser | NA | 32305225 |
| Exon 3 | c.607T>G | p. Tyr203Asp | NA | 32305225 |
| Exon 3 | c.610C>A | p. Arg204Ser | NA | NA |
| Exon 3 | c.610C>G | p. Arg204Gly | NA | 32305225 |
| Exon 3 | c.611G>C | p. Arg204Pro | NA | 32305225 |
| Exon 3 | c.628G>A | p. Gly210Ser | NA | 32305225 |
| Exon 3 | c.649T>A | p. Cys217Ser | HUA, Gout, CKD | NA |
| Exon 3 | c.649T>G | p. Cys217Gly | NA | 32305225 |
| Exon 3 | c.649T>C | p. Cys217Arg | NA | 32305225 |
| Exon 3 | c.665G>C | p. Arg222Pro | NA | 32305225 |
| Exon 3 | c.667T>G | p. Cys223Gly | HUA, Gout, HTN | NA |
| Exon 3 | c.668G>A | p. Cys223Tyr | NA | 32305225 |
| Exon 3 | c.674C>A | p. Thr225Lys | NA | 32305225 |
| Exon 3 | c.674C>T | p. Thr225Met | NA | 32305225 |
| Exon 3 | c.686T>G | p. Met229Arg | NA | 32305225 |
| Exon 3 | c.688T>C | p. Trp230Arg | NA | 32305225 |
| Exon 3 | c.706C>T | p. Pro236Ser | NA | 32305225 |
| Exon 3 | c.707C>A | p. Pro236Gln | HUA | 24096149 |
| Exon 3 | c.707C>T | p. Pro236Leu | NA | 32305225 |
| Exon 3 | c.707C>G | p. Pro236Arg | NA | 32305225 |
| Exon 3 | c.710C>G | p. Ser237Cys | NA | 32305225 |
| Exon 3 | c.736_756del | p. Lys246_Ser252del | NA | 32305225 |
| Exon 3 | c.739G>C | p. Arg247Pro | NA | NA |
| Exon 3 | c.744C>G | p. Cys248Trp | HUA, HTN, CKD | 24096149 |
| Exon 3 | c.749A>T | p. His250Leu | NA | 32305225 |
| Exon 3 | c.757G>T | p. Gly253Cys | NA | 32305225 |
| Exon 3 | c.761A>C | p. His254Pro | HUA, Gout, CKD | 38812211 |
| Exon 3 | c.764G>A | p. Cys255Tyr | HUA, CKD | 30072181 |
| Exon 3 | c.770T>C | p. Leu257Pro | NA | 32305225 |
| Exon 3 | c.772T>G | p. Trp258Gly | HUA, CKD | 31412356 |
| Exon 3 | c.774G>C | p. Trp258Cys | NA | 32305225 |
| Exon 3 | c.800G>T | p. Cys267Phe | NA | 32305225 |
| Exon 3 | c.805G>T | p. Gly269Cys | NA | 32305225 |
| Exon 3 | c.808G>T | p. Gly270Cys | NA | 32305225 |
| Exon 3 | c.814_816del | p. Tyr272del | NA | 32305225 |
| Exon 3 | c.817G>T | p. Val273Phe | HUA, Gout, CKD | 36290580 |
| Exon 3 | c.817G>C | p. Val273Leu | NA | 32305225 |
| Exon 3 | c.820T>C | p. Tyr274His | NA | 32305225 |
| Exon 3 | c.821A>G | p. Tyr274Cys | NA | 31544508 |
| Exon 3 | c.844T>C | p. Cys282Arg | NA | 32305225 |
| Exon 3 | c.844T>A | p. Cys282Ser | NA | 32305225 |
| Exon 3 | c.851T>C | p. Leu284Pro | NA | 32305225 |
| Exon 3 | c.854C>A | p. Ala285Glu | Gout, HUA | 25904457 |
| Exon 3 | c.857A>G | p. Tyr286Cys | NA | 32305225 |
| Exon 3 | c.860G>A | p. Cys287Phe | HUA, CKD | 33384418 |
| Exon 4 | c.890G>A | p. Cys297Tyr | NA | 32305225 |
| Exon 4 | c.891T>G | p. Cys297Trp | NA | 32305225 |
| Exon 4 | c.898T>G | p. Cys300Gly | NA | 32305225 |
| Exon 4 | c.898T>A | p. Cys300Ser | NA | 32305225 |
| Exon 4 | c.898T>C | p. Cys300Arg | HUA, Gout, CKD | NA |
| Exon 4 | c.943T>C | p. Cys315Arg | NA | 32305225 |
| Exon 4 | c.944G>A | p. Cys315Tyr | NA | 32305225 |
| Exon 4 | c.947A>C | p. Gln316Pro | HUA, CKD | 30072181 |
| Exon 4 | c.950G>A | p. Cys317Tyr | NA | 32305225 |
| Exon 4 | c.1039 T>C | p. Cys347Arg | HUA, CKD | 33384418 |
| Exon 5 | c.1153C>T | p. Arg385Trp | HUA, Gout, CKD | 25904457 |
| Exon 7 | c.1382C>A | p. Ala461Glu | NA | 32305225 |
| Exon 7 | c.1406C>T | p. Thr469Met | NA | 32305225 |
| Exon 7 | c.1462G>C | p. Gly488Arg | NA | 32305225 |
| Exon 7 | c.1499C>T | p. Ala500Val | HUA, CKD | This study |
| Exon 8 | c.1676A>G | p. Tyr559Cys | HUA, Gout, HTN, CKD | This study |
| Exon 8 | c.1648G > A | p. Val550Ile | hematuria | 31068128 |
| Exon 9 | c.1815A>G | p. Thr605Gly | HUA, HTN, CKD | 22907299 |

CKD: chronic kidney disease; HTN: hypertension; HUA: hyperuricemia; NA: not available.
